# *Notes from the Field:* Responding to the Wartime Spread of Antimicrobial-Resistant Organisms — Ukraine, 2022

**DOI:** 10.15585/mmwr.mm7249a5

**Published:** 2023-12-08

**Authors:** Ihor Kuzin, Oleksandr Matskov, Roman Bondar, Rostyslav Lapin, Tetiana Vovk, Andrea Howard, Arkadii Vodianyk, Robert Skov, Sarah Legare, Marianna Azarskova, Teeb Al-Samarrai, Ezra Barzilay, Charles Vitek

**Affiliations:** ^1^Ministry of Health of Ukraine, Kyiv, Ukraine; ^2^Public Health Center of the Ministry of Health of Ukraine, Kyiv, Ukraine; ^3^Clinical Center of Anesthesiology and Intensive Care, Vinnytsia M.I. Pirogov Regional Clinical Hospital, Vinnytsia, Ukraine; ^4^Ternopil Regional Clinical Hospital, Ternopil, Ukraine; ^5^Khmelnytskyi City Hospital, Khmelnytskyi, Ukraine; ^6^ICAP at Columbia University, New York, New York; ^7^Department of Epidemiology, Columbia University Mailman School of Public Health, New York, New York; ^8^Ukraine Country Office, World Health Organization, Kyiv, Ukraine; ^9^European Society of Clinical Microbiology and Infectious Diseases, Basel, Switzerland; ^10^Division of Global Health Protection, CDC Georgia; ^11^Division of Global HIV & Tuberculosis, Global Health Center, Office of the Director, CDC Ukraine; ^12^Division of Global Health Protection, Global Health Center, CDC; ^13^Office of the Director, Global Health Center, CDC Georgia.

Worldwide, bacterial antimicrobial resistance is estimated to cause more deaths than HIV or malaria and is recognized as a leading global public health threat (*1*). In Ukraine, the confluence of high prewar rates of antimicrobial resistance, an increase in the prevalence of traumatic wounds, and the war-related strain on health care facilities is leading to increased detection of multidrug-resistant organisms with spread into Europe (*2*,*3*). Evidence of increased rates of antimicrobial resistance in other conflict settings such as Iraq (*4*), and the long-term consequences for civilian, military, and other populations, argue that the spread of antimicrobial resistance in Ukraine is an urgent crisis that must be addressed, even during an ongoing war.

In mid-2022, a collaboration was established between CDC, the Center for Public Health of Ukraine (UPHC), local clinical and public health authorities, and international partners, including the World Health Organization regional office for Europe, ICAP at Columbia University, and the European Society for Clinical Microbiology and Infectious Diseases. The purpose of this collaboration was to improve laboratory detection, clinical treatment, and infection control response for antimicrobial resistance in the Ternopil, Khmelnytskyi, and Vinnytsia regions supported by U.S. Ukraine supplemental appropriations emergency funding.*

## Investigation and Outcomes

In August 2022, UPHC and regional collaborators conducted infection prevention and control and antimicrobial resistance laboratory capacity assessments in the three regional public health facilities and the three regional hospitals in the Ternopil, Khmelnytskyi, and Vinnytsia regions. This activity was reviewed by CDC, deemed not research, and was conducted consistent with applicable federal law and CDC policy.^†^

The infection prevention and control assessments identified inadequacies in surveillance of health care–associated infections, implementation of infection prevention and control measures such as recommended hand hygiene, and monitoring, evaluation, and feedback to the hospital staff members.^§^^,^^¶^ The laboratory assessments identified multiple challenges, especially inadequate quantities of automated microbiology equipment, and suboptimal laboratory quality and information management systems, biosafety practices, and staffing, as well as inconsistent availability of essential antibiotic susceptibility testing consumables. 

UPHC also conducted health care–associated infections and antimicrobial resistance point prevalence surveys at three regional hospitals during November–December 2022. Among 353 patients on surveyed wards, 50 (14%) had health care–associated infections.** High rates of antimicrobial resistance were identified among isolates from patients with health care–associated infections, with 30 of 50 (60%) patients having an infection with a carbapenem-resistant organism. Among 20 *Klebsiella pneumoniae* isolates, all were resistant to third-generation cephalosporins, and among the 19 *Klebsiella* isolates tested, all were also carbapenem-resistant. These rates are substantially higher than those reported from a 2016–2017 European Union–wide point prevalence survey, which included more than 300,000 acute care hospital patients and 100,000 long-term care facility residents; among these respondents, the study found a health care–associated infection rate of 5.5%. Among the subset of infections caused by the Enterobacteriaceae family of bacteria (including *Klebsiella*), 6.2% of isolates were resistant to carbapenem (*5*).

## Preliminary Conclusions and Actions

Urgent capacity building to prevent, detect, and respond to antimicrobial resistance is needed to save lives within Ukraine and limit international spread. UPHC and partners are collaborating to improve laboratory detection of antimicrobial resistance, antimicrobial prescribing, and infection prevention and control, starting in the Ternopil, Khmelnytskyi, and Vinnytsia regions. UPHC is prioritizing interventions to strengthen infection prevention and control and the laboratory-clinical interface via multidisciplinary hospital teams, establishing routine health care–associated infections and antimicrobial resistance surveillance, utilizing guidelines and locally collected data to inform clinical care, upgrading laboratory equipment and workflows, increasing availability and use of hand-hygiene disinfectants, and providing technical training for staff members. UPHC has issued clinical guidance on indications for bacteriology testing, including to military hospitals. Partners are supporting training curricula that include clinical and laboratory twinning^††^ between international experts on antimicrobial resistance and Ukrainian clinicians and laboratorians. In addition, partners are conducting workshops for regional and hospital staff members to develop and use clinical and laboratory standard operating procedures to strengthen infection prevention and control practices and clinical management of infected patients. Lastly, partners are working to provide additional laboratory supplies to meet the increased wartime demands, to capacitate laboratories to test for bacterial susceptibility to newer-generation antibiotics, and to improve reliable hospital access to these antibiotics.

To address the alarming increase of antimicrobial resistance in Ukraine, UPHC with assistance from international partners, is developing locally led and implemented measures to address antimicrobial resistance and will need ongoing support to scale them nationally. In addition, development of national action plans and context-specific policies and strategies are needed to improve infection prevention and control and monitor antibiotic use and antimicrobial resistance. 

## Footnotes

* The U.S. State Department’s Office of the Coordinator of U.S. Assistance to Europe and Eurasia transferred to CDC via an interagency agreement.

† 45 C.F.R. part 46.102(l)(2), 21 C.F.R. part 56; 42 U.S.C. Sect. 241(d); 5 U.S.C. Sect. 552a; 44 U.S.C. Sect. 3501 et seq.

§
https://www.cdc.gov/drugresistance/intl-activities/laarc.html

¶
https://www.who.int/publications/i/item/WHO-HIS-SDS-2018.9

**
https://www.ecdc.europa.eu/en/publications-data/point-prevalence-survey-healthcare-associated-infections-and-antimicrobial-use-4
